# Clinical and inflammatory factors associated with the extent of resection in primary, sporadic vestibular schwannomas: A retrospective study

**DOI:** 10.1186/s40478-025-02127-4

**Published:** 2025-10-03

**Authors:** Lorenz Dörner, Elisa-Maria Suhm, Vanessa Ries, Vitor Moura Goncalves, Marco Skardelly, Marcos Tatagiba, Jens Schittenhelm, Felix Behling

**Affiliations:** 1 Department of Neurosurgery and Neurotechnology, University Hospital Tübingen, Eberhard-Karls-University, Baden-Württemberg Tübingen, Germany Hoppe-Seyler-Street 3, 72076; 2 Center for Neuro-Oncology, Comprehensive Cancer Center Tübingen-Stuttgart, University Hospital Tübingen, Eberhard-Karls-University, Baden-Württemberg Tübingen, Germany; 3 Faculty of Medicine, University of Porto, Porto, Portugal; 4 Department of Neurosurgery, Klinikum am Steinenberg, Baden-Württemberg Reutlingen, Germany; 5 Department of Neuropathology, University Hospital Tübingen, Eberhard- Karls-University, Baden-Württemberg Tübingen, Germany

**Keywords:** Vestibular schwannoma, Acoustic neuroma, Inflammation, Tumor microenvironment, Macrophage, Lymphocyte, Extent of resection

## Abstract

**Background:**

The extent of resection (EOR) is known to impact recurrence free survival in vestibular schwannomas (VS). Identifying predictive factors for complete resection may direct treatment decisions in the future. In recent years there is increasing evidence for the involvement of inflammatory processes in the development and growth of VS. It is currently unclear whether inflammatory changes may also play a role in the extent of resection in VS.

**Methods:**

In this retrospective study, we analyzed clinical data, tumor extension, cystic characteristics and immunohistochemical markers for inflammation (CD68, CD163, CD3, CD8) and proliferation (MIB-1) as potential factors influencing the EOR in 1007 surgically treated primary sporadic VS. With CART-determined specific cut-offs for each inflammation marker, a common inflammatory score from 0 to 2 was determined. Univariate and multivariate analyses were performed for the EOR.

**Results:**

Total resection was achieved in 86.5% of cases. Incomplete resection was associated with advanced age (*p* = 0.0002), larger tumor size (*p* < 0.0001) and cystic characteristics on preoperative imaging (*p* < 0.0001). Increased expression of CD163, CD68 and CD3 (*p* < 0.0001, *p* = 0.0015 and *p* = 0.0024 respectively) was associated with partial tumor resection (PR). CD8 was significant when its CART-determined cut-off was considered (*p* = 0.0032). A higher inflammatory score was significantly connected to partial resection (*p* < 0.0001). In the multivariate analysis, larger size (*p* < 0.0001), older age (*p* = 0.0051), cystic characteristics (*p* = 0.0005) and higher CD68 expression (*p* = 0.0341) were independently significant factors for partial resection.

**Conclusions:**

Advanced age, greater tumor extension, cystic growth and higher infiltration with macrophages are independent factors for a less radical extent of resection.

**Supplementary Information:**

The online version contains supplementary material available at 10.1186/s40478-025-02127-4.

## Introduction

Vestibular schwannomas (VS) are the most common tumor entity of the cerebellopontine angle (CPA) with an annual incidence of approximately 1.52:100,000 [1]. Most VS occur sporadically, characterized by slow, indolent growth and a well-defined border [2, 3]. Unilateral sensorineural hearing impairment, vertigo, tinnitus and gait instability may occur as initial symptoms of patients with sporadic vestibular schwannomas [4, 5]. Facial or trigeminal nerve involvement can be observed in cases of larger tumors [6, 7]. Three main treatment strategies have been established. For smaller, stable and asymptomatic VS, watchful waiting with frequent MRI scans may be a possible option [8]. Microsurgical resection and radiotherapy are the main strategies for achieving tumor control in large, progressive, or symptomatic tumors [5, 8, 9]. Weighing the risks and benefits of each treatment modality is an ongoing challenge and frequently debated [5, 10].

For tumor control in VS, the extent of resection (EOR) is a strong predictive factor regarding recurrence-free survival [5, 11, 12]. To date, there is growing evidence suggesting that increased age and larger tumor size correlate with less radical resections [13]. In vestibular schwannoma surgery, dissection of the tumor from ambient structures like the brainstem and cranial nerves is critical. Tumor adherence to surrounding structures can complicate total extirpation due to preservation of critical structures and is associated with incomplete resection [14, 15].

Besides alterations in the NF2 gene on chromosome 22, which encodes for the tumor-suppressor protein merlin [16], the specific inflammatory tumor microenvironment (TME) is considered promoting tumorigenesis, growth and proliferation in sporadic VS [17–19]. The inflammatory TME is characterized by tissue infiltration with immune cells, particularly pro-tumoral M2-type (M2) tumor-associated macrophages (TAM) and lymphocytes, and the presence inflammatory mediators [20]. Former studies displayed the connection between the infiltration with tumor associated macrophage (TAM) and increased growth of VS [21, 22]. Further, the association between tumor adherence and increased tumor tissue inflammation has been displayed [14]. To better understand factors that influence the EOR in VS and thus patient prognosis, it seems important to consider the inflammatory TME. However, a comprehensive analysis of factors predicting the EOR in a large cohort of primary VS including tumor inflammation has not yet been conducted. Determining predictive factors for successful radical resection could help to guide future treatment strategies and facilitate postoperative prognostication of recurrence risk.

## Materials and methods

### Study cohort

All patients that were surgically treated in the authors’ institution between November 2003 and May 2019 were considered for this retrospective single center study (*n* = 1381). Cases were excluded due to missing consent for scientific use of tissue and clinical data (*n* = 68) and missing or insufficient tumor tissue (*n* = 25). Due to the sole analysis of primary VS, prior irradiated (*n* = 41) and recurrent (previously resected) (*n* = 57) cases were also excluded. Cases with missing clinical data (*n* = 14) and if the diagnosis of NF2-related schwannomatosis was present (*n* = 103) were excluded, resulting in 1073 cases for analysis.

**Fig. 1:**
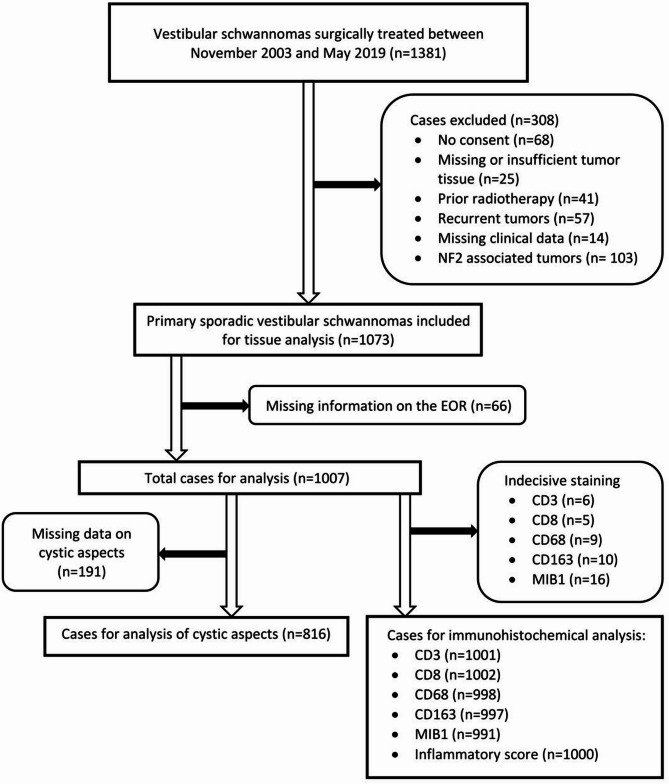
Flow diagram showing cases included and excluded

The following clinical data was collected by an electronic chart review: age, sex, tumor extension (Koos classification) and cystic characteristics on preoperative imaging. The diagnosis of cystic VS was based exclusively on the analysis of preoperative MRI data. The criteria employed to identify these lesions included the presence of either a solitary or multiple fluid-filled cavities within the tumor, in conjunction with the presence of solid components, and surrounded by a gadolinium-enhancing outer membrane. The extent of resection and the positioning was assessed according to the surgical reports. Postoperative MRI scans were not utilized in determining the extent of resection. To address a possible misclassification bias, the extent of resection was defined as follows: total resection (TR), meaning complete extirpation of the tumor with no residual; partial resection (PR) was defined as a residual tumor mass of any size, including capsule remnants, that had to be left in situ. The positioning for operation was documented as semi-sitting with a retrosigmoid approach or as “others”, when another position or approach was utilized. For the analysis of the extent of resection 66 further cases were excluded due to missing sufficient information necessary to categorize the radicality of surgery. This left 1007 cases for analysis of factors associated with the extent of resection (see Fig. 1).

### Tissue microarray construction and immunohistochemical staining

Vestibular schwannoma tissue samples from our institution’s neuropathological archive were used to construct tissue microarrays (TMA). Routine hematoxylin and eosin stains were reviewed, and several representative tumor areas were marked. Tumor sample cylinders measuring 1 mm in diameter were extracted from formalin-fixated and paraffin-embedded tumor tissue blocks according to the prior marking. For each tumor two sample cylinders were extracted with a conventional microarrayer (Beecher Instruments, Sun Prairie, Wisconsin, USA) and assembled in a chessboard manner on a recipient paraffin block. Cerebral and cerebellar cortex and a colorectal carcinoma metastasis were used as controls. Finalized TMAs were cut with a microtome to produce 4 micrometer slices that were subsequently dried (80 °C for 15 min) and immunohistochemically stained.

### Immunohistochemical staining

All immunohistochemical staining was done with an automated immunostainer (Ventana Medical Systems, Tucson, Arizona, USA). After pretreatment with Cell Conditioning Solution CC1 (pH 8.5), TMAs were incubated with the following antibodies: CD3 (1:500 at 42 °C, Thermo Fischer Scientific, Waltham, USA), CD8 (ready to use, at 37 °C, Roche, Basel, Switzerland), CD68 (1:200 at 42 °C, Agilent DAKO, Santa Clara, USA), CD163 (1:1000, at 37 °C, ABD Serotec, Puchheim, Germany), MIB1 (1:200, 42 °C, DAKO, Santa Clara, USA). OptiView HQ Universal Linker and OptiView Multimer were subsequently applied for 12 min each. Counterstaining was done with hematoxylin.

### Quantification and scoring of immunopositivity

For CD3 and CD8 immunopositive cells were counted in each cylinder resulting in immunopositive cells per 1 mm cylinder. For CD68 and CD163 a semi-quantitative score was applied, differentiating between negative samples (0), < 25% (1), 25–50% (2), 50–75% (3) and > 75% (4) immunopositivity.

Archived routine stainings for MIB1 that were done during the diagnostic tumor workup, were used. Images of areas with representative immunopositivity were taken of each tumor sample and processed for automated quantification with the Image J software (Version 1.51j8, NIH, Bethesda, 342 MD, USA) and the plugins Bio-Formats (Release 5.4.1; Open Microscopy Environment, 343 Madison, NJ, USA) and ImmunoRatio (Version 1.0c, Institute of Biomedical Technology, University of Tampere, Finland).

### Conception of the inflammatory score

For the common inflammatory score (IS) cut-off scores for the best dichotomization regarding total vs. partial resection were determined via classification and regression tree (CART) analyses for each inflammatory immunohistochemical marker (CD163, CD68, CD3 and CD8). CART-specific cut-offs were > 1 for CD163 and CD68 and $\:\ge\:$ 43.33 cells/mm, $\:\ge\:$ 31.11 cells/mm for CD8 and CD3, respectively. The common IS was defined as follows: Neither macrophage (CD163/CD68) nor lymphocyte (CD3/CD8) markers showed a score surpassing their cut-offs (IS = 0). Either macrophage or lymphocyte marker scores exceeded their cut-offs (IS = 1). Both macrophage and lymphocyte marker scores exceeded their cut-offs (IS = 2).

### Statistical analysis

The statistics software JMP^®^ version 17.2.0 (Cary, NC: SAS Institute Inc.; 1989) was applied for statistical analyses. CART analyses were conducted to divide continuous and ordinal parameters in relation to the EOR. Pooled t-tests and Pearson’s Chi-squared tests were done for continuous and ordinal/nominal parameters, respectively. To prevent overfitting, the scores for the inflammatory markers CD3, CD8, CD68, and CD163, along with the inflammatory score, were incorporated into the multivariate analysis as dichotomized variables. For multivariate analysis a binary logistic regression was done to identify independent factors associated with the extent of resection. For all statistical tests a significance level of α < 0.05 was used.

For assistance with grammar and style, the authors made intermittent use of the DeepL Write (Cologne, Germany; DeepL SE; 2017).

## Results

### Patient cohort characteristics

Our cohort showed a mean age of 48.9 years at surgery with a balanced female to male ratio (1.04). Most tumors had a larger extension in the cerebellopontine angle with T3 or T4 accounting for 38.8 and 34.8%, respectively while 22.4% reached T2 and 4.0% T1 according to the Koos classification. Cystic characteristics on preoperative MRI were observed in 8% of cases. In 82.5% of cases, the surgical positioning was semi-sitting with a retrosigmoid approach, while in the remaining cases, other approaches were utilized. Details are depicted in Table 1.

**Table 1:** Distribution of clinical and radiographic parameters and the inflammatory score. EOR, extent of resection; TR, total resection; PR, partial resection, percentage in parentheses

Sex	
Female Male	514 (51.0) 493 (49.0)
Mean age (in years)	48.8
Tumor extension (Koos)	
T1 T2 T3 T4 T1/2 T3/4	40 (4.0) 226 (22.4) 391 (38.8) 350 (34.8) 266 (26.4) 741 (74.6)
Cystic aspect on MRI (*n* = 816)	
Yes No	65 (8.0) 751 (92.0)
Positioning/approach	
Semi-sitting/retrosigmoid others	831 (82.5) 176 (17.5)
EOR	
TR PR	871 (86.5) 136 (13.5)
Inflammatory score (*n* = 1000)	
0 1 2	349 (34.9) 313 (31.3) 338 (33.8)

### Factors associated with the extent of resection

In 66 cases the description of the extent of resection in operative report was not detailed enough, leaving 1007 VS for the analysis of the extent of resection. A total resection was achieved in 86.5% and a partial resection in 13.5% of cases (for details see Table 1). No difference regarding sex was observed but patients which received a partial resection were significantly older (52.6 years) than patients who underwent a total resection (48.3 years, *p* = 0.0002). Tumors that were partially resected were more extensive in size, reaching T3/4 in 95.6% compared to 70.1% in radically resected VS (*p* < 0.0001). Cystic characteristics on preoperative MRIs were more commonly associated with partial resection (22.9%) than with complete resection (5.8%, *p* < 0.0001).

**Fig. 2:**
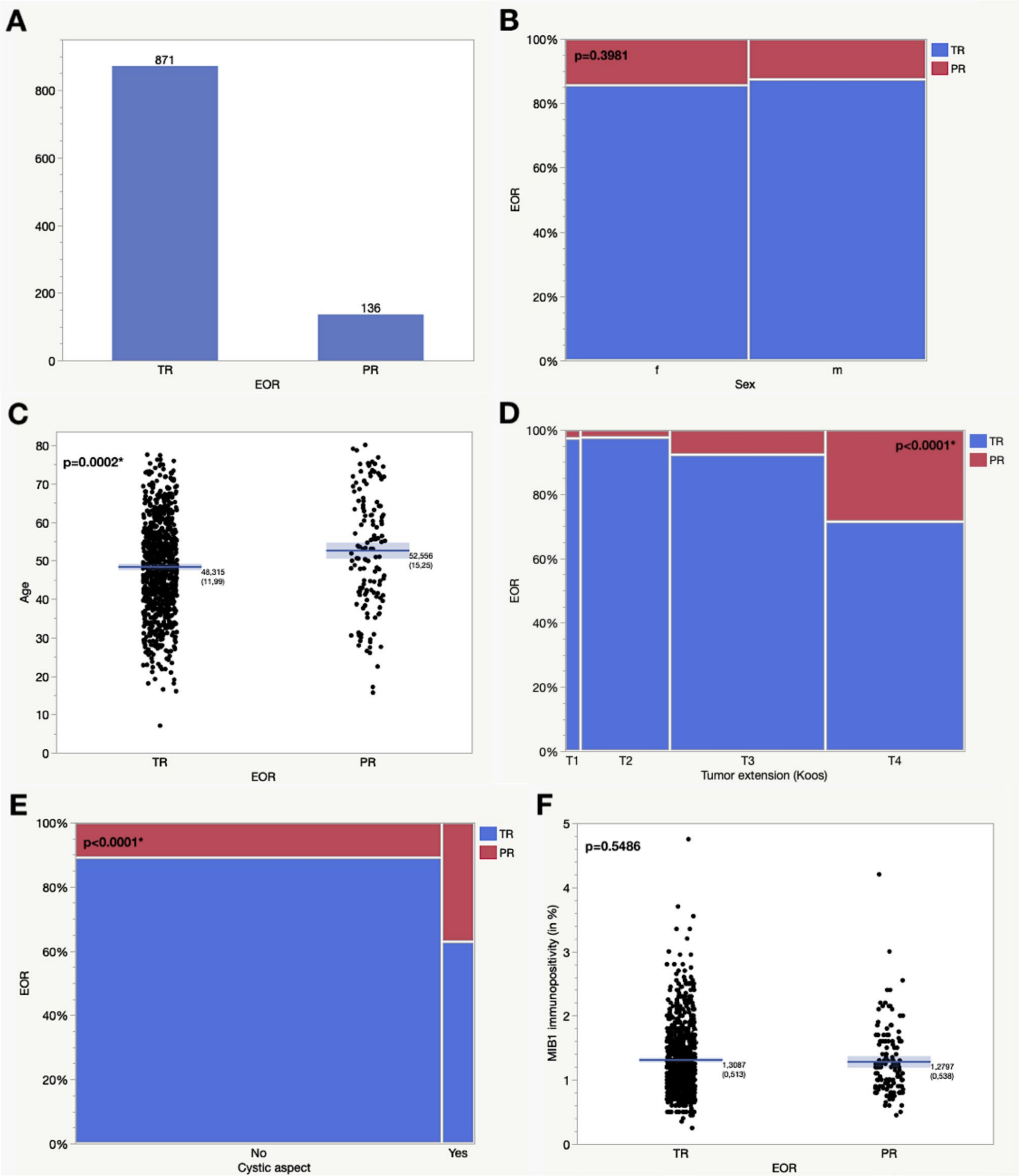
Total number of total resections and partial resections (**A**). Differences in the extent of resection in relation to sex (**B**), age (**C**), tumor extension (Koos) (**D**), cystic aspects on the preoperative MRI (**E**) and the MIB1-immunopositivity (**F**). EOR, extent of resection; TR, total resection; PR, partial resection

The expression of the proliferative marker MIB1 did not differ in the tumor tissue after partial (1.28%) or total resection (1.31%, *p* < 0.5486). The results are displayed in Fig. 2; Table 2.

**Table 2:** Differences in the extent of resection regarding clinical factors and the immunohistochemical expression of MIB1. TR, total resection; PR, partial resection, percentage in parentheses

Parameter	TR	PR	*p*-value (Prob > ChiSq/t-test)
Complete cohort (*n* = 1007)	871 (86.5)	136 (13.5)	-
Sex			
Female Male	440 (50.5) 431 (49.5)	74 (54.4) 62 (45.6)	0.3981
Mean age (in years)	48.3	52.6	0.0002*
Tumor extension (Koos)			
T1 T2 T3 T4	39 (4.5) 221 (25.4) 361 (41.4) 250 (28.7)	1 (0.7) 5 (3.7) 30 (22.1) 100 (73.5)	< 0.0001*
T1/2 T3/4	260 (29.9) 611 (70.1)	6 (4.4) 130 (95.6)	< 0.0001*
Cystic aspect on MRI (*n* = 816)			
Yes No	41 (5.8) 670 (94.2)	24 (22.9) 81 (77.1)	< 0.0001*
MIB1 immunopositivity (in %) (*n* = 991)	1.31	1.28	0.5486

### EOR and inflammation marker expression

Due to missing immunohistochemical expression data, 9, 10, 6 and 5 further cases had to be excluded for CD68, CD163, CD3 and CD8, respectively (see Fig. 1 for details).

Macrophage infiltration was more pronounced in cases with partial resection. A CD68 immunopositivity score of 2 or above ($\:\ge\:$ 25% of tumor tissue) was observed in 49.5% in complete resections compared to 64.7% in partial resections (*p* = 0.0010). For CD163 immunopositivity, a score of 2 or higher was reached in 42.7% of partially resected cases, in comparison to 23.5% in total resections (*p* < 0.0001). A similar difference was seen when comparing lymphocyte infiltration and the extent of resection. The mean immunopositivity count for CD3 was 37.26 ± 49.45 per mm biopsy cylinder in VS that were radically resected. Higher counts were seen in the tumor tissue after partial resections (51.33 count/mm, *p* = 0.0024). For the expression of CD8 (count/mm) no significant difference was observed. However, when the CART-specific cut-off ($\:\ge\:$ 43.33 count/mm) was considered, 44.9% of partially resected tumors exhibited a score exceeding the cut-off in comparison to 32.0% of total resections (*p* = 0.0032).

**Fig. 3:**
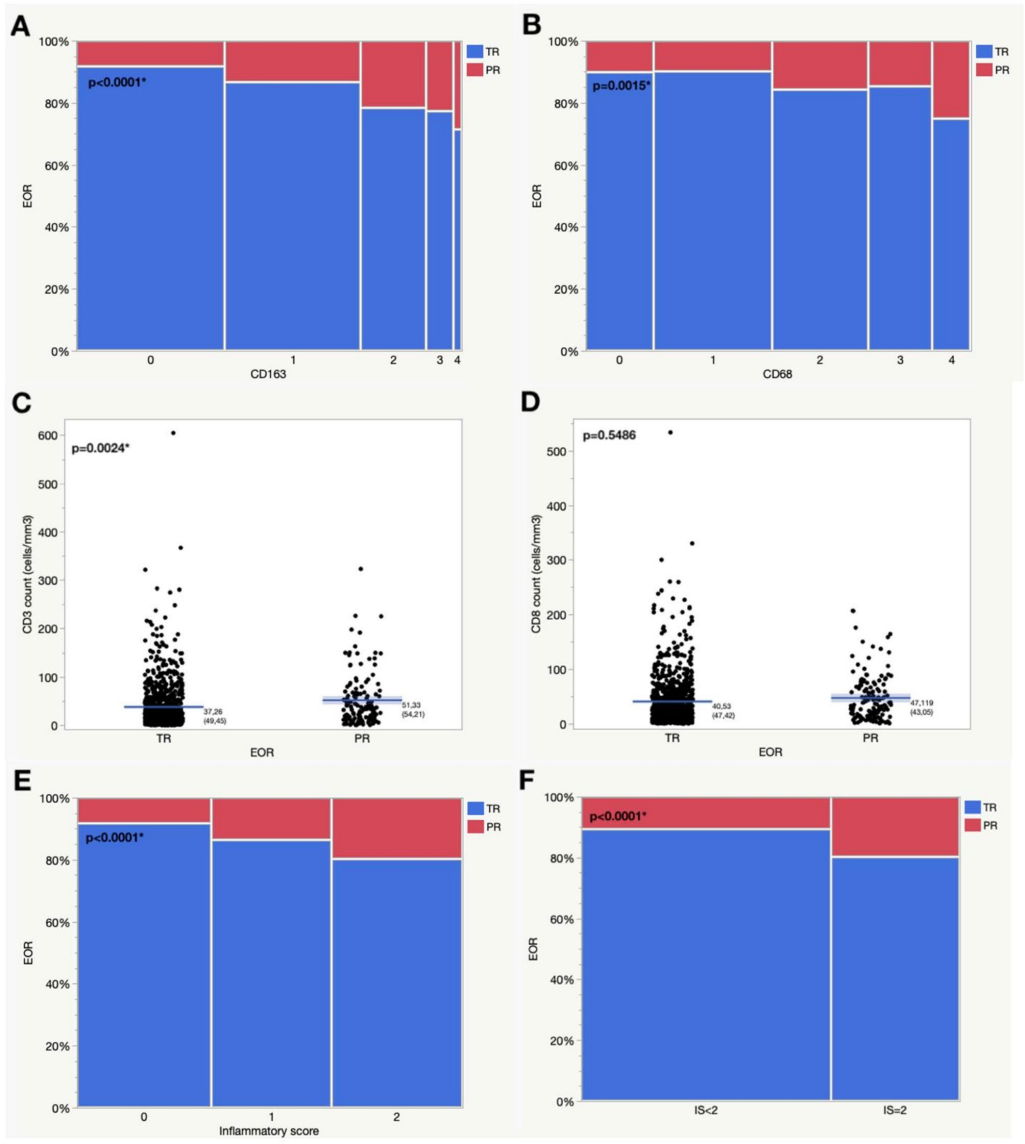
Differences in the extent of resection according to the expression of inflammation markers (CD163 (**A**), CD68 (**B**), CD3 (**C**) and CD8 (**D**). Differences in the inflammatory score (**E**) and the dichotomized inflammatory score (IS < 2, IS = 2) (**F**) according to the extent of resection. EOR, extent of resection; TR, total resection; PR, partial resection; IS, inflammatory score

A total of 1000 cases were considered for the inflammation score analysis. The distribution of the inflammatory score was balanced with 34.9% (0), 31.3% (1) and 33.8% (2) (see Table 1 for details). An inflammatory score of 2 was observed in 48.5% of partial resections and in 31.5% of total resections (*p* < 0.0001). The association between the EOR, inflammation markers and the inflammatory score is delineated in Fig. 3; Tables 3 and 4.

**Table 3:** Differences in the extent of resection in relation to the immunohistochemical expression of inflammation markers and the inflammatory score. TR, total resection; PR, partial resection

Parameter	TR *n* (%)	PR *n* (%)	*p*-value (Prob > ChiSq/t-test)
Complete cohort (*n* = 1007)	871 (86.5)	136 (13.5)	-
CD68 expression score (*n* = 998)			
0 1 2 3 4	160 (18.6) 275 (31.9) 210 (24.4) 142 (16.5) 75 (8.7)	18 (13.2) 30 (22.1) 39 (28.7) 24 (17.7) 25 (18.4)	0.0015*
</=1 >1	435 (50.5) 427 (49.5)	48 (35.3) 88 (64.7)	0.0010*
CD163 expression score (*n* = 997)			
0 1 2 3 4	354 (41.1) 305 (35.4) 132 (15.3) 55 (6.4) 15 (1.7)	31 (22.8) 47 (34.6) 36 (26.5) 16 (11.8) 6 (4.4)	< 0.0001*
</=1 >1	659 (76.5) 202 (23.5)	78 (57.3) 58 (42.7)	< 0.0001*
CD3 expression count/mm (*n* = 1001)			
< 31.11 >/=31.11	537 (62.1) 328 (37.9)	59 (43.4) 77 (56.6)	< 0.0001*
CD8 expression count/mm (*n* = 1002)			
< 43.33 >/=43.33	589 (68.0) 277 (32.0)	75 (55.1) 61 (44.9)	0.0032*
Inflammatory score (*n* = 1000)			
0 1 2	321 (37.1) 271 (31.4) 272 (31.5)	28 (20.6) 42 (30.9) 66 (48.5)	< 0.0001*
< 2 = 2	592 (68.5) 272 (31.5)	70 (51.5) 66 (48.5)	< 0.0001*

**Table 4:** Differences in the immunohistochemical expression of CD3 and CD8 regarding the extent of resection. CD3 and CD8 in count/mm. The p-value indicates the statistical significance of the differences between TR and PR for each marker. TR, total resection; PR, partial resection; SD, standard deviation; SE, standard error

	mean $\:\pm\:$ SD	SE	*p*-value (t-test)
CD3 (*n* = 1001)			0.0024*
TR	37.26 $\:\pm\:$ 49.45	1.68	
PR	51.33$\:\:\pm\:$ 54.21	4.65	
CD8 (*n* = 1002)			0.1277
TR	40.53 $\:\pm\:$ 47.42	1.61	
PR	47.12 $\:\pm\:$ 43.05	3.69	

### Multivariate analysis of the EOR

All clinical parameters that showed a significant association with the extent of resection in the univariate analysis were included in the multivariate binary logistic regressions.

For the inflammation markers, CART-specific cut-offs for the best dichotomization were applied and 799 cases were included in the multivariate analysis. Independent factors that were associated with a partial resection were identified. Larger tumor size (Koos T3/T4, *p* = 0.0002), cystic characteristics (*p* = 0.0005) and older age (*p* = 0.0080) were independent significant factors for partial resection. Regarding inflammation markers, only a CD68 score exceeding its cut-off score (> 1) was significant for PR. Sex, MIB1-, CD8-, CD3- and CD163-expression were without statistical significance (for details see Table 5).

An additional multivariate binary logistic regression with 803 cases was performed for the EOR including the designed inflammatory score excluding the individual inflammatory markers. The second analysis produced similar results. The most pronounced impact was seen by increased tumor extension expressed by T3/4 according to the Koos classification (*p* < 0.0001). Also, cystic characteristics were independent for achieving an incomplete resection (*p* = 0.0005). Older age was identified as another independent factor for decreased surgical radicality (*p* = 0.0051). In addition, an inflammatory score of 2 was independently predictive of partial resection (*p* = 0.0062). Sex and the expression of MIB1 were without independent significance in the multivariate model (for details see Table 6).

**Table 5:** Multivariate binary logistic regression for partial resection with IHC marker cut-offs (*n* = 799)

	Estimate (95% CI)	ChiSquare	*p*-value (Prob > ChiSq)
Intercept (TR)	-3.08(-4.33 – -1.86)	23.98	< 0.0001*
Sex (f)	-0.01 (-0.23–0.21)	0.01	0.9151
Age	0.02 (0.006–0.04)	7.02	0.0080*
Koos (T3/4)	0.89 (0.47–1.42)	14.00	0.0002*
Cystic characteristics	0.54 (0.23–0.84)	12.26	0.0005*
MIB1 expression (in %)	-0.20 (-0.67–0.23)	0.76	0.3828
CD163 (score) > 1	0.18 (-0.08–0.44)	1.94	0.1639
CD68 (score) > 1	0.28 (-0.02–0.54)	4.49	0.0341*
CD8 (count/mm) $\:\ge\:$43.33	-0.14 (-0.46–0.18)	0.78	0.3770
CD3 (count/mm) $\:\ge\:$31.11	0.24 (-0.08–0.57)	2.23	0.1356

**Table 6:** Multivariate binary logistic regression for partial resection with the inflammatory score (*n* = 803)

	Estimate (95% CI)	ChiSquare	*p*-value (Prob > ChiSq)
Intercept (TR)	-3.21(-4.43 – -2.03)	27.75	< 0.0001*
Sex (f)	0.002 (-0.22–0.22)	0	0.9830
Age	0.02 (0.008–0.04)	7.86	0.0051*
Koos (T3/4)	0.92 (0.51–1.46)	15.22	< 0.0001*
Cystic characteristics	0.52 (0.23–0.81)	12.25	0.0005*
MIB1 expression (in %)	-0.13 (-0.59–0.29)	0.37	0.5437
Inflammatory score (> 1)	0.31 (0.09–0.53)	7.49	0.0062*

## Discussion

With emerging evidence on VS management and evolving technology, a trend towards less invasive and radical treatment strategies can be observed [5, 23]. Less radical surgery is often performed to preserve hearing and facial nerve function [5, 24]. In general, the risk of recurrence must be weighed against the risk of cranial nerve affection. It is well established that less surgical radicality is associated with a decreased recurrence-free interval in patients with VS [11, 12, 25]. Therefore, it seems important to investigate factors that predict the extent of resection. Identifying possible factors may guide for or against surgery and may furthermore influence intraoperative decision making when aiming for radicality.

### EOR and clinical/radiographic characteristics

The results demonstrate that especially larger size, Koos T3 or T4, cystic characteristics as well as older age were independently predictive of incomplete tumor resection.

In the past, the correlation between higher age, greater tumor extension and a less radical extent of resection has been shown in studies on primary VS [12, 13, 26]. Macielak et al. pointed out, that not only older age and greater tumor extension may be of significance, but also cystic growth of VS is significantly connected to less surgical radicality [13]. Their study investigated 502 primary, sporadic VS located in the CPA and included subjective documentation by the surgeon and postoperative magnetic resonance imaging (MRI) scans to detect residual tumor [13].

A recent study from Scheer et al., which examined possible factors predicting progression and recurrence, pointed out similar correlations in 386 primary resected, sporadic VS [11]. Less radical resection was correlated with higher age and greater tumor extension (Koos) [11]. Macielak et al. performed additional postoperative MRI scans to assess the EOR [13], while Scheer et al. and our investigations on the EOR were based on surgical reports and surgeons’ impressions [11]. Regarding our large patient cohort, the findings of both studies were replicable. The elevated occurrence of incomplete resections in elderly patients may be ascribed to the presence of adhesions, which result in nearly total resections with the objective of preserving the cochlear and facial nerve. In the present study, the total resections and the capsule remnants resulting from partial resections were collectively considered. In addition, the increased incidence of incomplete resection rates may be attributable to a more conservative approach, characterized by an emphasis on preserving the facial nerve over a complete resection. This strategy is predicated on the objective of ensuring facial nerve function and minimizing morbidity [5, 27].

During VS surgery a challenge surgeons must encounter is the adherence of the tumor to surrounding cranial nerves and the brainstem. For total extirpation, it is critical to perform meticulous dissection, considering a potential affection of the cranial nerves. Cystic VS can exhibit adhesions which complicate dissection and impair surgical radicality [28, 29]. In 2024, Zhang et al. investigated the extent of resection and tumor adherence, cystic growth and inflammation in 28 primary sporadic VS [14]. In this study, cystic VS showed significantly more often adherences to the facial nerve and the brainstem (27%) when compared to solid VS (0%) [14]. However, no difference was observed for the EOR [14]. This may be due to the small number of 15 cystic cases observed in the study. Our data shows a significant independent negative impact of cystic characteristics on the EOR in a large cohort of primary VS. Concluding from these observations, tumor size, patient’s age and cystic characteristics in preoperative MRI scans may direct treatment decisions when aiming for total resection in patients with primary VS.

### EOR and inflammation

In our postoperative histopathological analyses, the immunohistochemical markers indicating macrophage infiltration (CD68 and CD163) and CD3 positive lymphocyte infiltration (CD3) were significantly linked to a lower extent of resection. Regarding the CART-specific cut-off for CD8 in the univariate analysis, high levels of CD8 positive lymphocyte infiltration were also significantly connected to less surgical radicality.

In the inflammatory microenvironment, two distinct macrophage phenotypes are differentiated. While M1-type macrophages are known to be pro-inflammatory and anti-tumoral, M2-type (CD163 positive) macrophages are characterized by regenerative, pro-proliferative and pro-tumoral characteristics [30].

It has been shown that larger VS and higher growth rates are associated with an increased expression of TAM markers [17, 21, 22]. Leisz and colleagues demonstrated heightened CD68 and CD163 expression levels in large VS and increased expression levels of CD68 in fast-growing VS [21]. Earlier, de Vries et al. found a significant correlation between the immunohistochemical expression of CD68 with tumor size and growth in 67 sporadic VS [17]. Later, de Vries and colleagues compared 10 fast growing and 10 stable VS and demonstrated a critical connection between accelerated growth and the expression of CD163, identifying M2 macrophages [22]. The results of these studies indicate that greater tumor extension, as shown in our previous study, is associated with increased TAM infiltration [31, 32]. Additionally, as aforementioned, tumor size predicts surgical radicality [13].

In our univariate and in the multivariate analysis, a higher expression of the marker CD68 was independently associated with higher rates of PR (see Table 5). Other inflammation markers (CD3, CD8 and CD163) were only significant in the univariate analyses. As mentioned above, impaired surgical radicality may be, besides other factors, the consequence of tumor adherence. It has been established that tumor tissue inflammation, specifically the presence of M1 and M2 macrophages, plays a role in the development of peritumoral adhesions [14, 19]. Pro-tumoral M2 macrophages stimulate angiogenesis, cell proliferation and tissue remodeling through the secretion of pro-proliferative cytokines of the advanced phase of inflammation [30, 33]. An important mediator of fibroblast activation and tissue remodeling is the transforming growth factor $\:\beta\:$ (TGF$\:\beta\:$) family [34]. Macrophage secretion of TGF$\:\beta\:$ and basic fibroblast growth factor activates fibroblasts in the TME and the tumor capsule [35]. With modulation of the TME, tumor associated fibroblasts can promote tumor progression, the recruitment of anti-inflammatory cells and the polarization of macrophages towards their M2 phenotype and T lymphocytes towards Th2 cells [36]. For tissue remodeling and organ fibrosis, Th2 cells play a critical role. Collagen production in the extracellular matrix by fibroblasts is directly stimulated by Th2 cell secreted interleukins (IL) 4, IL5 and IL13 [34]. Increased activation of tumor associated fibroblasts through M2 macrophage and Th2 cells might thus lead to fibrosis and peritumoral adhesions to surrounding structures. Additionally, M2 macrophages are capable of inhibiting cytotoxic (CD8 positive) T-lymphocytes through immune checkpoints [30]. Further, the recruitment and differentiation of immunosuppressing and pro-tumoral regulatory T(reg) lymphocytes can be induced by M2 macrophage secreted TGF$\:\beta\:$ [30, 37]. Besides other mechanisms, T(reg) lymphocytes mediate immunosuppression by secretion of TGF$\:\beta\:$[38] and subsequent effects of TGF$\:\beta\:$, as mentioned above, can be promoted.

A recent study conducted by Duhon et al. investigated on biomechanical stiffness and preoperative magnetic resonance elastography (MRE) to assess objective tumor stiffness in sporadic VS and meningiomas [15]. Their findings suggest that heightened vestibular schwannoma stiffness is connected to subtotal resections and poorer clinical outcomes [15]. Further, VS stiffness significantly correlated with higher CD163 + macrophage infiltration, a higher intensity of alpha smooth muscle actin, a marker for activated fibroblasts, and an increased presence of interdispersed collagen [15].

The multivariate analysis, delineated in Table 5, indicates that tumor tissue infiltration with CD68 positive macrophages is required for a significant impact on the EOR. However, the inflammatory score, might be solely driven by the significant impact of the expression of CD68 marker in partially resected VS. A former study by Zhang et al. showed a significantly higher expression of CD163 in adherent VS [14]. However, they also found that the marker CD80, which is expressed on M1 macrophages [39], was also significantly elevated in adherent VS [14]. Perry et al. investigated M1/M2 macrophage infiltration in 46 subtotally resected sporadic VS [33]. They demonstrated that infiltration of M1 macrophages was associated with unfavorable outcomes and worse tumor control [33]. Considering the findings of our study, which underscore the role of the pan-macrophage marker CD68, it can be concluded that the intricacies of the specific TME are multifaceted and not solely governed by M2-type macrophages. Clearly, macrophage infiltration predicts a less radical resection.

### Clinical implications

In the management of vestibular schwannomas, one must consider multiple factors including clinical symptoms, growth rate and tumor size, age and patients’ treatment preferences. The decision to pursue or avoid microsurgical resection for alternative treatment strategies must be done on a case-by-case basis. The identification of predictors for a partial resection of primary sporadic vestibular schwannomas may facilitate the development of a more precise and personalized therapeutic decision-making in the future. When aiming for a radical resection, the patients’ age, tumor size and cystic characteristics on preoperative MRI scans should be considered. Moreover, our study identified macrophage infiltration as a predictor for incomplete tumor resection. Thus, a high-risk group for an incomplete resection was detected. Analyzing inflammatory markers may aid in identifying cases with higher recurrence risk. Certain players in in the TME are often discussed as possible therapeutic targets [19]. Targeting TAM mediated inflammation preoperatively and therefore modify growth, stiffness and the formation of tumor adhesions could enable for higher rates of successful resections and more favorable outcomes [14, 15].

### Limitations

The cohort consisted of solely surgically treated sporadic VS, therefore the inevitable selection bias represents one major limitation. Further, the study was retrospective. The EOR was subjectively assessed by the surgeons’ intraoperative impression and may differ between institutions. However, VS surgery in our center is performed by few experienced senior surgeons with a uniform approach to intraoperative evaluation of radicality. A recent comparative study by Mahboubi et al. displayed, that the assessment of residual tumors should be done by early postoperative MRI scans and the surgeons’ impression seems to be rather inaccurate in case of a near-total resection [40]. On the other hand, small tumor remnants may not be discernable from applied muscle patches in the internal acoustic meatus, making the intraoperative impression by the surgeon invaluable. Moreover, 66 cases were excluded due to missing data concerning the EOR, and 191 cases were excluded due to missing radiologic data on cystic characteristics. The investigator’s approach was not dictated by any particular parameter. Missing EOR data can be attributed to the inaccuracy or incompleteness of surgical reports. In the event of missing data concerning cystic characteristics, this is since MRI assessment of cystic characteristics was not performed on the most recent cases, including the 191. We conducted a series of comparative analyses, including univariate and multivariate methods, to assess the differences between cases that were included and those that were excluded from the study. These analyses can be seen in supplementary Tables 1–4, which compare the included cases and the cases with missing data on the EOR and cystic characteristics, respectively. With respect to the comparison of included cases with those lacking EOR data, the missing EOR data group was found to be significantly smaller in size (T1/2) and exhibited reduced macrophage marker expression in the univariate analysis. The multivariate analysis did not yield a statistically significant result. Excluded cases (missing cystic data) compared to included cases showed higher macrophage marker expression and slightly lower MIB1 expression when compared to the included cases in the univariate analysis. In the multivariate analysis, higher MIB1 and CD3 expression was found to be protective against exclusion, while a higher CD163 expression was associated with exclusion.

Given the specific scope of our study, which focused on the extent of resection, the investigation did not extend to the assessment of surgical complications, such as infections. Additionally, the examination did not encompass the evaluation of hearing and facial nerve function.

A future study with the primary focus on tumor inflammation and recurrence-free survival, as well as postoperative morbidity, would be a worthwhile endeavor.

In addition, a promising avenue for future exploration could involve an investigation into facial nerve impairment and hearing function.

Due to small numbers of incomplete resections, we solely differentiated between total and partial resection, which includes near total and subtotal resection. Despite that, our large cohort of 1007 VS included relevant numbers of partially resected VS and cystic VS. A multivariate analysis for the EOR was performed and a clear set of independent factors were identified. Nevertheless, a future study of a similar sample size and an EOR evaluation through postoperative MRI may be required to substantiate our findings. Moreover, the surgical intent may be evaluated in conjunction with this additional investigation.

## Conclusions

This study identified advanced age, greater tumor extension, cystic growth, and higher infiltration with macrophages as independent factors associated with less radical resection.

## Supplementary Information

Below is the link to the electronic supplementary material.

Supplementary Material 1

Supplementary Material 2

Supplementary Material 3

Supplementary Material 4

## Data Availability

No datasets were generated or analysed during the current study.

## Abbreviations

CD3: Cluster of differentiation 3
CD8: Cluster of differentiation 8
CD68: Cluster of differentiation 68, CD163:Cluster of differentiation 163
CPA: Cerebellar pontine angle
EOR: Extent of resection
MIB1: Mindbomb E3 ubiquitin protein ligase 1
MRI: Magnetic resonance imaging
PR: Partial resection
SD: Standard deviation
SE: Standard error
TAM: Tumor associated macrophage
TGF$\:\beta\:$: Transforming growth factor$\:\beta\:$
TME: Tissue microenvironment
TR: Total resection
VS: Vestibular Schwannoma
