# Supplementary material for: Clinical and inflammatory factors associated with the extent of resection in primary, sporadic vestibular schwannomas: A retrospective study
Source: Acta Neuropathol Commun. 2025 Oct 3;13:211. doi: 10.1186/s40478-025-02127-4 (PMC12492732; doi:10.1186/s40478-025-02127-4)
Supplement: Supplementary file 1 — Supplementary Material 1 [file 40478_2025_2127_MOESM1_ESM.docx]

| Parameter | Included  n (%) | Excluded (no EOR data)  n (%) | p-value  (Prob>ChiSq/t-test) |
| --- | --- | --- | --- |
|  | 1007 (93.9) | 66 (6.1) | - |
| Sex  Female  Male | 514 (51.0)  493 (49.0) | 36 (54.6)  30 (45.4) | 0.5813 |
| Mean age (in years) | 48.89 | 45.86 | 0.0590 |
| Tumor extension (Koos)  T1  T2  T3  T4  T1/2  T3/4 | 40 (4.0)  226 (22.4)  391 (38.8)  350 (34.8)  266 (26.4)  741 (73.6) | 9 (13.6)  16 (24.2)  26 (39.4)  15 (22.7)  25 (37.9)  147 (62.1) | 0.0015*  0.0424* |
| Cystic characteristics (n=877)  cystic  solid | 65 (8.0)  751 (92.0) | 4 (6.6)  57 (93.4) | 0.6935 |
| MIB1 expression (in %, n=530) | 1.30 | 1.23 | 0.2693 |
| CD68 expression (score, n=1063)  0  1  2  3  4  </=1  >1 | 178 (17.8)  305 (30.6)  249 (25.0)  166 (16.6)  100 (10.0)  483 (48.4)  515 (51.6) | 20 (30.8)  23 (35.4)  12 (18.5)  6 (9.2)  4 (6.1)  43 (66.1)  22 (33.9) | 0.0407*  0.0055* |
| CD163 expression (score, n=1062)  0  1  2  3  4  </=1  >1 | 385 (38.6)  352 (35.3)  168 (16.9)  71 (7.1)  21 (2.1)  737 (73.9)  260 (26.1) | 33 (50.8)  23 (35.4)  7 (10.8)  2 (3.1)  0 (0)  56 (86.1)  9 (13.9) | 0.1660  0.0280* |
| CD3 expression (count/mm, n=1066)  <31.11  >/=31.11 | 39.17  596 (59.5)  405 (40.5) | 33.25  43 (66.1)  22 (33.9) | 0.3513  0.2917 |
| CD8 expression (count/mm, n=1067)  <43.33  >/=43.33 | 41.42  596 (59.5)  406 (40.5) | 35.77  40 (61.5)  25 (38.5) | 0.3390  0.7432 |
| Inflammatory score (n=1065)  0  1  2  <2  =2 | 339 (33.9)  309 (30.9)  352 (35.2)  648 (64.8)  352 (35.2) | 27 (41.5)  23 (35.4)  15 (23.1)  50 (76.9)  15 (23.1) | 0.1331  0.0436* |

**Supplementary Table 1** Differences between included and excluded (missing data on the extent of resection) cases regarding clinical and immunohistochemical parameters. TR, total resection; PR, partial resection; IS, inflammatory score, percentage in parentheses
