# Supplementary material for: Clinical and inflammatory factors associated with the extent of resection in primary, sporadic vestibular schwannomas: A retrospective study
Source: Acta Neuropathol Commun. 2025 Oct 3;13:211. doi: 10.1186/s40478-025-02127-4 (PMC12492732; doi:10.1186/s40478-025-02127-4)
Supplement: Supplementary file 2 — Supplementary Material 2 [file 40478_2025_2127_MOESM2_ESM.docx]

**Supplementary Table 4** Binary logistic regression for exclusion (no cystic data) vs. inclusion.PR, partial resection; EOR, extent of resection

|  | Estimate (95% CI) | ChiSquare | p-value (Prob>ChiSq) |
| --- | --- | --- | --- |
| Intercept (included) | -0.14(-2.99 – -0.09) | 0.09 | 0.7599 |
| Sex (f) | -0.04 (-0.25 – 0.29) | 0.23 | 0.6303 |
| Age | -0.003 (-0.04 – 0.01) | 0.23 | 0.6343 |
| Koos (T3/4) | 0.04 (-0.48 – 0.11) | 0.17 | 0.6835 |
| EOR (PR) | 0.03 (-0.53 – 0.60) | 0.06 | 0.8038 |
| MIB1 expression (in %) | -0.76 (-0.93 – 0.23) | 15.71 | <0.0001* |
| CD163 (score) >1 | 0.71 (-0.53 – 0.33) | 43.21 | <0.0001* |
| CD68 (score) >1 | -0.02 (-0.59 – 0.04) | 0.05 | 0.8230 |
| CD8 (count/mm) $\geq$43.33 | 0.03 (-0.08 – 0.75) | 0.06 | 0.8013 |
| CD3 (count/mm) $\geq$31.11 | -0.29 (-0.75– 0.13) | 3.99 | 0.0458* |
