# Supplementary material for: Clinical and inflammatory factors associated with the extent of resection in primary, sporadic vestibular schwannomas: A retrospective study
Source: Acta Neuropathol Commun. 2025 Oct 3;13:211. doi: 10.1186/s40478-025-02127-4 (PMC12492732; doi:10.1186/s40478-025-02127-4)
Supplement: Supplementary file 3 — Supplementary Material 3 [file 40478_2025_2127_MOESM3_ESM.docx]

| Parameter | Included  n (%) | Excluded (no cystic data)  n (%) | p-value  (Prob>ChiSq/t-test) |
| --- | --- | --- | --- |
|  | 816 (81.0) | 191 (19.0) | - |
| Sex  Female  Male | 419 (51.3)  397 (48.7) | 95 (49.7)  77 (50.3) | 0.6887 |
| Mean age (in years) | 48.96 | 48.58 | 0.7098 |
| Tumor extension (Koos)  T1  T2  T3  T4  T1/2  T3/4 | 35 (4.3)  187 (22.9)  318 (39.0)  276 (33.8)  222 (27.2)  594 (72.8) | 5 (2.6)  39 (20.4)  73 (38.2)  74 (38.7)  44 (23.0)  147 (77.0) | 0.4541  0.2394 |
| EOR  TR  PR | 711 (87.1)  105 (12.9) | 160 (83.8)  31 (16.2) | 0.2209 |
| MIB1 expression (in %, n=530) | 1.33 | 1.20 | 0.0023* |
| CD68 expression (score, n=998)  0  1  2  3  4  </=1  >1 | 154 (19.0)  249 (30.7)  209 (25.8)  125 (15.4)  74 (9.1)  403 (49.7)  408 (50.3) | 24 (12.8)  56 (30.0)  40 (21.4)  41 (21.9)  26 (13.9)  80 (42,8)  107 (57.2) | 0.0192*  0.0882 |
| CD163 expression (score, n=997)  0  1  2  3  4  </=1  >1 | 346 (42.7)  288 (35.5)  118 (14.6)  44 (5.4)  14 (1.7)  634 (78.3)  176 (21.7) | 39 (20.9)  64 (34.2)  50 (26.7)  27 (14.4)  7 (3.7)  103 (55.1)  84 (44.9) | <0.0001*  <0.0001* |
| CD3 expression (count/mm, n=1001)  <31.11  >/=31.11 | 39.49  482 (59.3)  331 (40.7) | 37.78  114 (60.6)  74 (39.4) | 0.6774  0.7336 |
| CD8 expression (count/mm, n=1002)  <43.33  >/=43.33 | 40.96  486 (59.7)  328 (40.3) | 43.41  110 (58.5)  78 (41.59 | 0.5194  0.7639 |
| Inflammatory score (n=1000)  0  1  2  <2  =2 | 285 (35.1)  247 (30.4)  281 (34.6)  532 (65.4)  281 (34.6) | 54 (28.9)  62 (33.1)  71 (38.0)  116 (62.0)  71 (38.0) | 0.2738  0.3794 |

**Supplementary Table 3** Differences between included and excluded (missing data on cystic characteristics) cases regarding clinical and immunohistochemical parameters. TR, total resection; PR, partial resection; EOR, extent of resection; IS, inflammatory score, percentage in parentheses
