# Supplementary material for: Clinical and inflammatory factors associated with the extent of resection in primary, sporadic vestibular schwannomas: A retrospective study
Source: Acta Neuropathol Commun. 2025 Oct 3;13:211. doi: 10.1186/s40478-025-02127-4 (PMC12492732; doi:10.1186/s40478-025-02127-4)
Supplement: Supplementary file 4 — Supplementary Material 4 [file 40478_2025_2127_MOESM4_ESM.docx]

**Supplementary Table 2** Binary logistic regression for excluded (missing data on extent of resection) vs. included. PR, partial resection

|  | Estimate (95% CI) | ChiSquare | p-value (Prob>ChiSq) |
| --- | --- | --- | --- |
| Intercept (included) | -1.52(-2.99 – -0.09) | 4.24 | 0.0394* |
| Sex (f) | 0.02 (-0.25 – 0.29) | 0.02 | 0.9015 |
| Age | -0.01 (-0.04 – 0.01) | 1.89 | 0.1695 |
| Koos (T3/4) | -0.19 (-0.48 – 0.11) | 1.54 | 0.2152 |
| Solid tumor | -0.025 (-0.53 – 0.60) | 0.01 | 0.9289 |
| MIB1 expression (in %) | -0.31 (-0.93 – 0.23) | 1.09 | 0.2973 |
| CD163 (score) >1 | -0.07 (-0.53 – 0.33) | 0.11 | 0.7351 |
| CD68 (score) >1 | -0.27 (-0.59 – 0.04) | 2.71 | 0.0994 |
| CD8 (count/mm) $\geq$43.33 | 0.34 (-0.08 – 0.75) | 2.56 | 0.1094 |
| CD3 (count/mm) $\geq$31.11 | -0.31 (-0.75– 0.13) | 1.85 | 0.1736 |
